# Who are the patients who use non-pharmacological home remedies? Cross-sectional study in Switzerland and France

**DOI:** 10.1093/fampra/cmae030

**Published:** 2024-05-27

**Authors:** Paul Sebo, Neria E Winkler, Marie Morel, Mohamed Amir Moussa, Dagmar M Haller, Hubert Maisonneuve

**Affiliations:** University Institute for Primary Care (IuMFE), University of Geneva, 1211 Geneva, Switzerland; University Institute for Primary Care (IuMFE), University of Geneva, 1211 Geneva, Switzerland; Department of Cardiology, University Heart Center Zurich, University Hospital Zurich, 8091 Zurich, Switzerland; University College of General Medicine, University Claude Bernard Lyon 1, 69008 Lyon, France; University Institute for Primary Care (IuMFE), University of Geneva, 1211 Geneva, Switzerland; University Institute for Primary Care (IuMFE), University of Geneva, 1211 Geneva, Switzerland; University Institute for Primary Care (IuMFE), University of Geneva, 1211 Geneva, Switzerland; University College of General Medicine, University Claude Bernard Lyon 1, 69008 Lyon, France

**Keywords:** France, home remedy, patient characteristic, primary care, survey, Switzerland, woman

## Abstract

**Background:**

Many patients may be tempted to use non-pharmacological home remedies (NPHRs) to relieve various complaints. To the best of our knowledge, there is little data on the characteristics of patients using NPHRs. In this cross-sectional study carried out between March 2020 and July 2021, we examined the socio-demographic factors underlying their use in patient populations in Switzerland and France.

**Methods:**

Using official registries, we randomly selected 50 primary care physicians (PCPs) in Geneva (Switzerland) and Lyon/Grenoble (France). Seven research assistants consecutively recruited patients from PCP waiting rooms (20–25 patients per practice). Patients completed a paper-based questionnaire assessing the use [yes/no] of 304 NPHRs for 79 medical conditions. The NPHR list was developed by our team with input from 97 patients. We used univariable and multivariable logistic regressions, adjusting for intra-cluster correlations, to examine associations between NPHR use and patient characteristics (gender, age, practice location, nationality, education level, and self-rated health).

**Results:**

Of the 1198 eligible patients, 1012 agreed to participate (85%). Overall, 635 patients (63%) reported using at least one of the remedies tested in the study. In multivariable analysis, women (OR = 1.7 [95%CI = 1.3–2.3], *P*-value < 0.001), younger patients (< 40 years: OR = 2.1 [95%CI = 1.6–2.9], *P*-value < 0.001), and French patients (OR = 1.6 [95%CI = 1.1–2.3], *P*-value < 0.001) tended to use NPHRs more often than other patients.

**Conclusions:**

Many patients, particularly women, young people, and French patients, reported using NPHRs. This survey’s findings hold the potential to inform healthcare providers, policymakers, and researchers about the diverse preferences that shape patients’ healthcare choices.

**Key messages**

- Non-pharmacological home remedy (NPHR) use is common in primary care patients.
- Use appears particularly more common in female and younger patients.
- Based on the literature, the most commonly used NPHRs appear to be safe and cheap.

## Introduction

In the ever-evolving landscape of medical practice, the use of traditional remedies remains a fascinating aspect of healthcare-seeking behavior [1]. Traditional remedies are also referred to as grandmother’s remedies in specific cultures in Europe [2] and India [3], or non-pharmacological home remedies (NPHRs) in Western cultures. These age-old remedies, rooted in cultural traditions and indigenous knowledge, encompass a spectrum of natural ingredients and practices believed to have therapeutic value [4]. Many patients continue to turn to these remedies to meet their health and well-being needs, despite advances in modern medicine [5, 6].

The aim of this study was to examine the socio-demographic factors underlying the use of NPHRs among patient populations in Switzerland and France, and to document the potential side effects and estimated cost of NPHRs.

## Methods

### Setting and participant recruitment

This cross-sectional survey was carried out between March/2020 and July/2021 in two neighboring geographical regions, Switzerland (canton of Geneva) and France (Lyon and Grenoble, encompassing departments of Rhône/Ardèche/Loire/Isère/Savoie/Haute-Savoie). This study was a component of a broader research initiative conducted within our research group, focusing on the use of NPHRs in primary care [5–10].

Our sampling framework was constructed using professional registries of primary care physicians (PCPs) from the three regions (Geneva/Lyon/Grenoble). We randomly selected 50 PCPs for inclusion in the study. Contact was established with these physicians via phone or email. In cases of refusal or non-response after three attempts, alternative PCPs were drawn from the list until 50 agreed to participate. A total of 168 PCPs were contacted (participation rate = 29.8%).

Seven research assistants recruited consecutive patients from the waiting rooms of the selected PCPs. Approximately 20–25 patients were recruited from each practice. Patient recruitment days were agreed in advance between research assistants and PCPs and patient recruitment took place on a single day. Eligibility criteria excluded patients <18 years, those lacking proficiency in French, and those seeking immediate medical attention. The Research Ethics Committee of Geneva granted approval for the study (Project-ID 2020-00939).

### Data collection

In a self-administered paper questionnaire completed in the waiting room (Supplementary Material 1 for the English version), patients were asked whether in the last 12 months, they had used one or more of the remedies on a list of 304 NPHRs for 79 medical conditions. We developed the list of NPHRs by consulting a random sample of 97 patients from 11 medical practices and refined it through discussions within our research team to finalize the list used in the study.

### Definition of NPHRs

As there is no universal consensual definition of NPHRs, we defined them in our study as remedies that are not available in the form of commercial medicines and do not require the help of an external therapist. Examples include honey for sore throats, thyme infusions for coughs, and rice cooking water for diarrhea.

### Sample size determination and data analysis

We calculated the sample size for an expected average prevalence of NPHR use of 60% with a 95%CI of ±5%. Our calculation accounted for clustering within medical practices (ICC = 0.05, average of 20 patients per PCP). A minimum of 720 participants was required, but the decision was made to recruit 1000 patients to accommodate potential missing data.

Descriptive statistics entailed proportions for categorical variables and medians with interquartile ranges (IQRs) for continuous variables. We examined the association between at least one NPHR used and patient characteristics [gender, age, practice location (rural/urban), nationality (French, Swiss, other), education level (university or equivalent, other), self-estimated health status (excellent/very good, good, moderate/poor)] using univariable/multivariable logistic regressions. We also examined the association between the number of NPHRs used and patient characteristics using univariable/multivariable binomial negative regressions. All regressions were adjusted for intra-cluster correlations. Statistical significance was set at a two-sided *P*-value of ≤0.05, and all data analyses were performed using STATA15.1 (College Station/TX).

### Side effects and cost of commonly used NPHRs

We conducted literature review using PubMed to identify and synthesize information on the side effects of the 10 most commonly used NPHRs in our population, as listed in a recent study using the same dataset [10]. We also estimated the cost of these remedies for Switzerland (in Swiss Francs) and France (in Euros), by consulting several shop websites (1 Euro = 1 Swiss Franc on 15/September/2023). Data extraction was done in duplicate (NW/PS). Discrepancies were resolved through discussion within the research team.

## Results

Of the 1198 eligible patients, 1012 agreed to participate (participation rate = 85%, women = 61%, median age = 52 years). Table 1 summarizes the participants’ main characteristics.

**Table 1.** Patients’ characteristics (*N* = 1012 patients).

Characteristic	*N* (%)	Median [IQR]
Female gender (*N* = 1008)	616 (61.1)	
Age (*N* = 1006)		52 [31]
Region (*N* = 1012)		
Grenoble (France)	360 (35.6)	
Lyon (France)	345 (34.1)	
Geneva (Switzerland)	307 (30.3)	
Location of the PCP’s medical practice (*N* = 1012)		
Urban zone	602 (59.5)	
Rural zone	410 (40.5)	
Nationality (*N* = 1009)		
French	690 (68.4)	
Swiss	219 (21.7)	
Other	100 (9.9)	
Marital status (*N* = 1004)		
Married or living as a couple	588 (58.6)	
Single	217 (21.6)	
Divorced or separated	127 (12.6)	
Widowed	72 (7.2)	
Work status (*N* = 1009)		
Occupational activity	493 (48.9)	
Retired	324 (32.1)	
Student or apprenticeship/ vocational training	61 (6.1)	
Recipient of unemployment (ALV) or invalidity (DI) benefits ^1^	55 (5.5)	
Other	764 (7.4)	
Completed training (*N* = 1009)		
University, FIT, UAS ^2^	354 (35.1)	
Intermediate school ^3^	479 (47.5)	
Compulsory schooling or no training/education	176 (17.4)	
Self-estimated general health status (*N* = 1007)		
Excellent or very good	319 (31.7)	
Good	524 (52.0)	
Moderate or poor	164 (16.3)	
Number of daily medications (*N* = 995)		1 [13]
Number of visits to the PCP in the past twelve months (*N* = 1010)		2 [0]

^1^ALV = unemployment insurance; DI = disability insurance.

^2^FIT = Federal Institute of Technology; UAS = University of Applied Sciences.

^3^Apprenticeship, vocational training, baccalaureate or diploma from intermediate school.

A little less than two-thirds of patients (*n* = 635) reported using at least one NPHR (median number of remedies used = 7, IQR = 22). Table 2 shows the association between NPHR use and patient characteristics. In multivariable analysis, women, younger patients, and French patients tended to use NPHRs more often and to use more NPHRs than other patients. For example, the odds of using NPHRs were 1.7 times higher for women than men (OR = 1.7[95%CI = 1.3–2.3], *P*-value < 0.001). Similarly, the incidence rate for the number of NPHRs used was 20% higher for women than for men (incident rate ratio = 1.2[95%CI = 1.1–1.4], *P*-value < 0.001).

**Table 2.** Associations between the use of non-pharmacological home remedies and patients’ characteristics (*n* = 1012 patients).

Characteristic	Unadjusted OR for use of at least one remedy from a list of 304 NPHRs (95%CI)^1^	*P*-value	Adjusted OR for use of at least one remedy from a list of 304 NPHRs (95%CI)^2^	*P*-value	Unadjusted IRR for number of remedies used (95%CI)^3^	*P*-value	Adjusted IRR for number of remedies used (95%CI)^4^	*P*-value
Gender		<0.001		<0.001		<0.001		<0.001
Female	1.7 (1.3–2.3)		1.7 (1.3–2.3)		1.2 (1.1–1.4)		1.2 (1.1–1.4)	
Male	1		1		1		1	
Age [years]		<0.001		<0.001		<0.001		<0.001
<40	2.1 (1.6–2.8)		2.1 (1.6–2.9)		1.3 (1.2–1.5)		1.3 (1.2–1.5)	
40–59	1.8 (1.3–2.5)		1.9 (1.4–2.6)		1.3 (1.1–1.4)		1.3 (1.1–1.4)	
≥ 60	1		1		1		1	
Location		0.26		0.38		0.25		0.36
Urban zone	1.2 (0.9–1.5)		1.1 (0.9–1.4)		1.1 (1.0–1.2)		1.0 (1.0–1.1)	
Rural zone	1		1		1		1	
Nationality		0.003		0.001		0.01		0.004
French	1.7 (1.2–2.3)		1.6 (1.1–2.3)		1.2 (1.1–1.4)		1.2 (1.0–1.4)	
Swiss	1		1		1		1	
Other	1.0 (0.7–1.6)		0.9 (0.5–1.4)		1.0 (0.8–1.2)		1.0 (0.8–1.2)	
Completed training		0.07		0.18		0.07		0.22
University, FIT, UAS^5^	1.3 (1.0–1.8)		1.2 (0.9–1.7)		1.1 (1.0–1.2)		1.1 (1.0–1.2)	
Other	1		1		1		1	
Self-estimated general health status		0.06		0.46		0.05		0.49
Excellent or very good	1.3 (1.1–1.6)		1.1 (0.9–1.4)		1.1 (1.0–1.2)		1.0 (1.0–1.1)	
Good	1		1		1		1	
Moderate or poor	1.0 (0.7–1.3)		1.2 (0.8–1.6)		1.0 (0.9–1.1)		1.1 (0.9–1.2)	

^1^Univariable logistic regression (adjusted for intra-cluster correlations within medical practices); OR = odds ratio.

^2^Multivariable logistic regression (adjusted for intra-cluster correlations within medical practices and for all variables listed in the table), OR = odds ratio.

^3^Univariable negative binomial regression (adjusted for intra-cluster correlations within medical practices); IRR = incident rate ratio.

^4^Multivariable negative binomial regression (adjusted for intra-cluster correlations within medical practices and for all variables listed in the table); IRR = incident rate ratio.

^5^FIT = Federal Institute of Technology; UAS = University of Applied Sciences.

Supplementary Material 1 lists the known side effects and the estimated cost of the 10 most commonly used NPHRs [10]. Side effects were generally rare and mild (e.g. risk of flatulence with prunes used to treat constipation). However, there were rare reports of temporary peripheral nerve palsy when ice/cold pads were used for contusions, hypothermia when cold water rinse was used for burns, and exposure to *Clostridium botulinum* spores in babies when honey was used. The estimated cost of these NPHRs was low, well under 1 Swiss Franc or 1 Euro per day.

## Discussion

### Main findings

In summary, we found that women, younger patients, and French patients tended to use NPHRs more often and to use more NPHRs than other patients. We also found that the reported side effects for NPHRs were mostly mild and that NPHRs were inexpensive.

### Comparison with existing literature

The association between the female gender and the use of NPHRs was also reported in previous studies [4, 11, 12]. In both Western and non-Western societies, sociological studies observed an association between female gender and the ability to care for others. Judi Aubel, an anthropologist specializing in community health and education programs, analyzed that in non-Western societies grandmothers play a predominant role in caring for women and children [13, 14]. She found that, although grandmothers’ knowledge and practices in terms of care were specific to their culture, the roles they played in their families were universal. These roles ranged from counseling to the direct provision of care to women and children and could be associated with the gendered aspects of the term “grandmother remedies” [13, 15].

Our study took place in Geneva and two neighboring French regions. This area presents a relatively homogenous environment with significant cross-border interactions, which may explain the low disparity in NPHR use. Further investigation into country-specific factors and cultural nuances could help discern more subtle differences in NPHR use between France and Switzerland.

We hypothesize that the increasing ecological conscience in younger generations may explain a higher interest for more “natural” remedies in younger age groups. Yet this hypothesis, as well as any other reason for the observed associations, requires further exploration. Nevertheless, our findings highlight the nuanced interplay between socio-demographic attributes and healthcare choices.

In addition to the fact that many NPHRs are considered by patients to be effective, as we showed in a previous study [10], the potential risks associated with their use are relatively low and they are generally inexpensive, which may contribute to their popularity among patients. Data on the safety and economic aspects of NPHRs could help healthcare providers and policymakers understand the overall impact and attractiveness of these traditional remedies in patient care.

### Limitations

Although our study benefited from a substantial sample size and encompassed three French-speaking European regions, caution is warranted when generalizing the results to other countries within Europe or beyond. Indeed, the diversity in NPHRs used can exhibit significant variations based on geographical and cultural influences. The age distribution of our population, with a slight overrepresentation of younger patients, may also impact the generalizability of our findings. This may be due to a reduced presence of older patients in practices during the COVID-19 epidemic and/or a reluctance among older patients to participate, due to their stronger reliance on conventional healthcare practices. The COVID-19 pandemic may have shifted patients’ healthcare-seeking behaviors, potentially leading to increased use of NPHRs as patients may have been hesitant to visit healthcare facilities. Finally, as is characteristic of any questionnaire-based observational study, the potential for memory bias introduces an element of uncertainty into our results.

## Conclusion

This cross-sectional study illuminates the socio-demographic dimensions of patients’ engagement with NPHRs in Switzerland and France, and contribute to the broader discourse on patient-centered healthcare and the integration of traditional practices into modern medical paradigms. These insights can inform healthcare providers and practitioners in tailoring healthcare interventions to meet the diverse preferences of patients. Our study also highlights the safety and affordability of NPHRs, two factors that certainly contribute to their widespread use among patients.

## Supplementary data

Supplementary material is available at *Family Practice* online.

cmae030_suppl_Supplementary_Material

## Data Availability

The data underlying this article will be shared on reasonable request to the corresponding author.
