# Supplementary material for: Who are the patients who use non-pharmacological home remedies? Cross-sectional study in Switzerland and France
Source: Fam Pract. 2024 May 27;41(5):841–5. doi: 10.1093/fampra/cmae030 (PMC11461152; doi:10.1093/fampra/cmae030)
Supplement: cmae030_suppl_Supplementary_Material [file cmae030_suppl_supplementary_material.pdf]

Table. Top 10 Non-Pharmacological Home Remedies (NPHRs) used by 1012 patients consulting primary care physicians in France and Switzerland, and their side effects, as reported in the literature, and estimated cost

NPHR	Health problem	Number of patients using the NPHR[1] (%) [95% CI]	Side effects (reference)[2]	Study design in cited reference, number of side effects	Estimated cost[3]
Ice / cold pad	Contusion	306 (30.2) [26.4 – 34.3]	Temporary peripheral nerve palsy (1) (2) (3) (4)<br>Frostbite injury (4)	(1) Review, no figures (rare event)<br>(2) Case report (n=5)<br>(3) Case report (n=6)<br>(4) Review, no figures (rare event)	EUR[4] 2.00<br>CHF[4] 2.50
Cold water rinse	Burn	297 (29.4) [25.3 – 33.7]	Hypothermia (5)	(5) Review, no figures (rare event)	N/A
Rice / rice cooking water	Diarrhoea	291 (28.8) [25.4 – 32.4]	None reported (6)	(6) Review, N/A	EUR[5] 0.06<br>CHF[5] 0.18
Honey / honey gargle	Sore throat	264 (26.1) [23.0 – 29.4]	Risk of exposure to *Clostridium botulinum* spores in children <12 months (7) (8)	(7) Review, no figures (rare event)<br>(8) Review, no figures (rare event)	EUR[6] 0.04<br>CHF[6] 0.075
Honey	Common cold	260 (25.7) [22.4 – 29.3]	Risk of exposure to *Clostridium botulinum* spores in children <12 months (7) (8)	(7) Review, no figures (rare event)<br>(8) Review, no figures (rare event)	EUR[6] 0.04<br>CHF[6] 0.075
Honey	Cough	249 (24.6) [21.5 – 28.1]	Risk of exposure to *Clostridium botulinum* spores in children <12 months (7) (8)	(7) Review, no figures (rare event)<br>(8) Review, no figures (rare event)	EUR[6] 0.04<br>CHF[6] 0.075
Walk (going for a stroll)	Low morale	240 (23.7) [20.5 – 27.3]	No severe side effects (9)<br>Minor side effects (muscle or joint pain, headache and fatigue) (9)	(9) Review, no figures	EUR[7] 0.25 – 0.50<br>CHF[7] 0.30 – 0.60
Prunes / plums	Constipation	227 (22.4) [19.7 – 25.4]	None reported (10) (11) (12)<br>Risk of flatulence (13) (14)	(10) Trial, N/A<br>(11) Review, N/A<br>(12) Review, N/A<br>(13) Trial (n=120), mean days per week = 4.7 (SD 2.7) intervention group vs 3.0 (SD 2.8) for control group<br>(14) Trial (n=54), mean days per week = 2.1 (range 0-7) for prune period vs 1.4 (range 0-7) for baseline	EUR[8] 0.30<br>CHF[8] 0.60
Heat (hot water bottle or hot shower)	Back pain	215 (21.3) [18.6 – 24.1]	No severe side effects (15) (16) (17)<br>Temporary redness in the area of heat application (16)	(15) Trial (n=61), N/A<br>(16) Trial (n = 371), n=1 out of 113 patients<br>(17) Review, N/A	N/A
Walk (going for a stroll)	Stress	209 (20.7) [17.7 – 23.9]	None reported (18)<br>No severe side effects (19)	(18) Trial (n=247), N/A<br>(19) Review, N/A	EUR[7] 0.25 – 0.50<br>CHF[7] 0.30 – 0.60

[1] Number of patients who reported using the NPHR for the indicated health problem in our study.
[2] Side effects reported in the literature.

[3] In Euros (EUR) for France, in Swiss Francs (CHF) for Switzerland.
[4] Estimated cost per unit. For a treatment lasting one week, the cost per day is therefore estimated at EUR 0.30 and CHF 0.35.
[5] Based on an estimated cost of EUR 1 and CHF 3 for a 1kg pack of rice in France and Switzerland, respectively, and 60g per dose/serving.
[6] Based on an estimated cost of EUR 8 and CHF 15 for a 1kg pot of honey in France and Switzerland, respectively, and 5g (a coffee spoon) per dose/serving.
[7] Based on an estimated cost of EUR 50 and CHF 60 for sports or running shoes in France and Switzerland, respectively, with a lifetime of 1000 km. Assuming an average distance of 5-10 km per walk, the estimated cost per walk is EUR 0.25 to 0.50 in France and CHF 0.30 to 0.60 in Switzerland, respectively.
[8] Based on an estimated cost of EUR 6 and CHF 12 for a 1kg dried plums in France and Switzerland, respectively, and 50g per dose/serving.

References

1. Mac Auley DC. Ice therapy: how good is the evidence? Int J Sports Med. 2001;22(5):379-84.
2. Drez D, Faust DC, Evans JP. Cryotherapy and nerve palsy. Am J Sports Med. 1981;9(4):256-7.
3. Malone TR, Engelhardt DL, Kirkpatrick JS, Bassett FH. Nerve injury in athletes caused by cryotherapy. J Athl Train. 1992;27(3):235-7.
4. Swenson C, Swärd L, Karlsson J. Cryotherapy in sports medicine. Scand J Med Sci Sports. 1996;6(4):193-200.
5. Daigeler A, Kapalschinski N, Lehnhardt M. [Therapy of burns]. Chirurg. 2015;86(4):389-401.
6. Gottlieb T, Heather CS. Diarrhoea in adults (acute). BMJ Clin Evid. 2011;2011.
7. DeGeorge KC, Ring DJ, Dalrymple SN. Treatment of the Common Cold. Am Fam Physician. 2019;100(5):281-9.
8. Werner A, Laccourreye O. Honey in otorhinolaryngology: When, why and how? European Annals of Otorhinolaryngology, Head and Neck Diseases. 2011;128(3):133-7.
9. Heissel A, Heinen D, Brokmeier LL, Skarabis N, Kangas M, Vancampfort D, et al. Exercise as medicine for depressive symptoms? A systematic review and meta-analysis with meta-regression. Br J Sports Med. 2023;57(16):1049-57.
10. Attaluri A, Donahoe R, Valestin J, Brown K, Rao SS. Randomised clinical trial: dried plums (prunes) vs. psyllium for constipation. Aliment Pharmacol Ther. 2011;33(7):822-8.
11. Lever E, Cole J, Scott SM, Emery PW, Whelan K. Systematic review: the effect of prunes on gastrointestinal function. Aliment Pharmacol Ther. 2014;40(7):750-8.
12. Katsirma Z, Dimidi E, Rodriguez-Mateos A, Whelan K. Fruits and their impact on the gut microbiota, gut motility and constipation. Food & Function. 2021;12(19):8850-66.
13. Lever E, Scott SM, Louis P, Emery PW, Whelan K. The effect of prunes on stool output, gut transit time and gastrointestinal microbiota: A randomised controlled trial. Clin Nutr. 2019;38(1):165-73.
14. Piirainen L, Peuhkuri K, Bäckström K, Korpela R, Salminen S. Prune juice has a mild laxative effect in adults with certain gastrointestinal symptoms. Nutrition Research. 2007;27(8):511-3.
15. Stark J, Petrofsky J, Berk L, Bains G, Chen S, Doyle G. Continuous low-level heatwrap therapy relieves low back pain and reduces muscle stiffness. Phys Sportsmed. 2014;42(4):39-48.
16. Nadler SF, Steiner DJ, Erasala GN, Hengehold DA, Hinkle RT, Beth Goodale M, et al. Continuous Low-Level Heat Wrap Therapy Provides More Efficacy Than Ibuprofen and Acetaminophen for Acute Low Back Pain. Spine. 2002;27(10):1012-7.
17. Hall H, McIntosh G. Low back pain (acute). BMJ Clin Evid. 2008;2008.
18. Awick EA, Ehlers DK, Aguiñaga S, Daugherty AM, Kramer AF, McAuley E. Effects of a randomized exercise trial on physical activity, psychological distress and quality of life in older adults. General Hospital Psychiatry. 2017;49:44-50.
19. Churchill R, Teo K, Kervin L, Riadi I, Cosco TD. Exercise interventions for stress reduction in older adult populations: a systematic review of randomized controlled trials. Health Psychol Behav Med. 2022;10(1):913-34.

Physician code: **Participant code:**

Survey on the use of « home remedies » by adult patients

Please read and sign the patient information form before completing this survey.

What do we think a « home remedy » is?
It is a remedy that has not been sold as a drug, as a product that acts on your health or with the intention of curing. A **«** home remedy » does not require the help of a therapist (e.g. physiotherapist, osteopath, homeopath, naturopath, herbalist, or more generally any certified healthcare professional) to be used. A « home remedy » does not include alternative and complementary medicine, but rather represents little things we do in our daily lives for self-care, without consulting a specialist.

① Have you used any « home remedies » in the last twelve months?

☐ Yes.

☐ No *(Please indicate the reason(s) by ticking the appropriate box(es) below) :*
- ☐ I don't know any « homes remedies ».
- ☐ I believe that « homes remedies » are ineffective.
- ☐ I have easy access to healthcare and don't need to take « homes remedies ».
- ☐ I prefer to consult my doctor rather than take « homes remedies ».
- ☐ I prefer using pharmaceutical drugs rather than « homes remedies ».
- ☐ I find « homes remedies » too expensive.
- ☐ Other *(Please specify.)* : ____

Part 1: Socio-demographic variables associated with patients

② Please indicate if your place of residence is a:

☐ City or urban area ☐ Semi-rural area ☐ Rural area

③ How many times have you visited your primary care physician in the last 12 months, including today's visit?

☐ Once ☐ 2 to 5 times ☐ 6 to 9 times ☐ 10 times or more

④ Overall, do you think your health is:

☐ Excellent ☐ Very good ☐ Good ☐ Fair ☐ Poor

⑤ How many pharmaceutical treatments do you usually take each day?

Please indicate the number of drug(s) taken per day: ______________________

⑥ Are you…:

☐ A man ☐ A woman ☐ I prefer not to reply.

⑦ What is your nationality?

☐ Swiss ☐ French ☐ German ☐ Italian ☐ Spanish ☐ Portuguese ☐ English

☐ Other *(Please specify.)*: ____________________________________

⑧ What is your year of birth?

19__________ 20__________

⑨ What is your family situation?

☐ Single ☐ Married/living as a couple ☐ Divorced/separated ☐ Widowed

☐ With dependent child(ren) ☐ Without dependent child(ren)

⑩ What is your current main professional situation (only one answer possible)?

☐ Student or trainee
☐ Farmer
☐ Craftsman, trader or business manager
☐ Executive
☐ Employee
☐ Laborer
☐ Intermediary profession
☐ Housewife/-man
☐ Unemployed
☐ Disabled (i.e. unable to work)
☐ Not working, neither unemployed nor unable to work
☐ Retired
☐ Other *(Please specify.)*: __________________________________

⑪ What is the highest level of education you have completed?

- ☐ Primary school or without diploma
- ☐ General Certificate of Secondary Education
- ☐ General baccalaureate
- ☐ Professional baccalaureate
- ☐ Higher education, baccalaureate + 3 years
- ☐ Higher education, baccalaureate + 5 years
- ☐ Higher education, baccalaureate + 8 years or more

⑫ What social security coverage do you have?

- ☐ Social security only
- ☐ Social security + mutual insurance
- ☐ Social security + additional health insurance
- ☐ State Medical Aid (SMA) ☐ Whitout ☐ Application in progress: __________

If you use home remedies and ticked « YES » to the first question (on page 1), please answer the questions in Part 2 and 3. If you do not use home remedies and ticked « NO » to the first question, you can stop here.

Part 2: Variables associated with the use of « homes remedies » in general

⑬ Have you talked to your primary care physician about your use of « homes remedies »?

- ☐ Yes (Please specify; multiple answers possible):
  - ☐ Yes, I raised the topic spontaneously.
  - ☐ Yes, my doctor raised the topic spontaneously.
  - ☐ Other *(Please specify.)* : ____
- ☐ No (Please specify; multiple answers possible):
  - ☐ No, because my physician did not ask me.
  - ☐ No, because I didn't feel the need to tell him/her.
  - ☐ No, because I forgot to tell him/her.
  - ☐ No, my physician raised the subject, but I didn't want to discuss it with him/her.
  - ☐ No, because I'm afraid of being misunderstood by my physician.
  - ☐ No, because I fear my physician's judgement in relation to this practice.
  - ☐ No, because I feel it is a self-care practice.
  - ☐ No, because this practice is not part of medical care.
  - ☐ Other *(Please specify.)* : ____

⑭ Do you think that the role of the primary care physician is to inform you about « homes remedies »?

- ☐ No, that is not his/her role.
- ☐ Yes, that is his/her role. (In this case, please specify if you prefer the subject to be raised...) :
  - ☐ spontaneously, without any specific request from my side.
  - ☐ only following a request from my side.

⑮ **For what reason(s) do you use « homes remedies »? (several answers possible)?**

- ☐ To avoid or delay a visit to the physician.
- ☐ Because I can treat myself or heal/get better by myself.
- ☐ For preventive purposes to stay healthy or to avoid getting sick.
- ☐ When the cost of medical care or pharmacological treatment seems too great for the health problem that I suffer from.
- ☐ Because I live or work in an area where it is difficult for me to see a physician.
- ☐ Because my physician did not prescribe any pharmacological treatment.
- ☐ In order to act as a complement to pharmacological treatment.
- ☐ In combination with pharmacological treatment, as the maximum dose was reached.
- ☐ As an alternative to conventional medicine. (If so, please specify; multiple answers possible.):
  - ☐ Because an effective pharmacological treatment doesn't exist.
  - ☐ To avoid side effects associated with pharmacological treatment(s).
  - ☐ To limit the number of pharmacological treatments taken.
  - ☐ Because I distrust pharmacological treatments.
  - ☐ Because I distrust conventional medicine.

Part 3: « Homes remedies » used

Think about what you do on a daily basis for your health, but also what you use frequently when you are sick. This can be plants, techniques, exercises, use of simple objects...

Note: this « homes remedy » has not been sold as a drug, as a product acting on your health or with the intention to cure. A « home remedy » does not require the assistance of a therapist (e.g. physiotherapist, osteopath, homeopath, naturopath, herbalist, or more generally any certified healthcare) to be used. A « home remedy » does not include alternative and complementary medicine, but rather represents little things that we do in our daily lives for self-care, without consulting a specialist.

Please indicate the « homes remedies » used by ticking the appropriate box(es) on the attached list.

Note: Magnetizers and “coupeurs de feu” (*i.e., common traditional healers in the region in which this study was undertaken*) can be considered as « homes remedies » if they practice within the private circle, without financial benefits).

Physician code: Participant code:

It is not necessary to fill in every line of the questionnaire, please only fill in the lines corresponding to the « home remedies » you have used in the last twelve months.

1. Part of the body affected.
2. Condition for which you have used a « home remedy ».
3. The remedy used / Please note that it is not necessary to complete steps 4, 5, 6 and 7 if you have not used these « homes remedies ».
4. If you are affected by any of the following conditions, please indicate how often you have used the remedy (all of the time, often, sometimes, rarely)
5. Specify the effectiveness of the remedy (ineffective, somewhat effective, moderately effective, very effective). If you are unable to comment, please tick "I don't know".
6. If you add honey and/or lemon to your remedy, tick the appropriate box(es).
7. Estimate the approximate cost of the remedy in Euros.
8. If you have used remedies not listed below, you can indicate them at the end of the questionnaire and tick the corresponding boxes for frequency, effectiveness, addition of honey and/or lemon, and specify the cost.

(1) **2. *SKIN AND EYE***

(3) ⚠	(4) Frequency of use				(5) Effectiveness							(7) Cost
	All of the time	Often	Sometimes	Rarely	Ineffective	Somewhat	Moderately	Very	I don't know	Lemon	Honey	€
2.6 Burn (2)												
Lily flower (plant to be applied)	☐	☐	☐	☐	☐	☐	☐	☐	☐	☐	☐	
Cold water rinse (to be applied)	☐	☐	☐	☐	☐	☐	☐	☐	☐	☐	☐	
Toothpaste (to be applied)	☐	☒	☐	☐	☐	☐	☒	☐	☐	☐	☐	2
Potato (to be applied)	☒	☐	☐	☐	☐	☒	☐	☐	☐	☐	☐	4
Butter (to be applied)	☐	☐	☐	☐	☐	☐	☐	☐	☐	☐	☐	
Vinegar (to be applied)	☐	☐	☐	☐	☐	☐	☐	☐	☐	☐	☐	
Aloe vera (plant to be applied)	☐	☒	☐	☐	☐	☐	☐	☒	☐	☐	☐	15
(8) ***11. HAVE YOU USED ANY OTHER HOME REMEDIES? IF SO, WHICH ONES?***		5										
	☐	☐	☐	☐	☐	☐	☐	☐	☐	☐	☐	

	Frequency of use				Effectiveness							Cost
	All of the time	Often	Sometimes	Rarely	Ineffective	Somewhat	Moderately	Very	I don't know	Lemon	Honey	€
**1. *HEART AND BLOOD CIRCULATION***												
1.1 Cholesterol												
Cider vinegar (to be swallowed)	☐	☐	☐	☐	☐	☐	☐	☐	☐	☐	☐	
Cloves + dried apricots (to be swallowed)	☐	☐	☐	☐	☐	☐	☐	☐	☐	☐	☐	
Rapeseed oil (to be swallowed)	☐	☐	☐	☐	☐	☐	☐	☐	☐	☐	☐	
Herbal tea (to be swallowed)	☐	☐	☐	☐	☐	☐	☐	☐	☐	☐	☐	
1.2 Hypertension												
Black garlic (to be swallowed)	☐	☐	☐	☐	☐	☐	☐	☐	☐	☐	☐	
Infusion of olive tree leaves (to be swallowed)	☐	☐	☐	☐	☐	☐	☐	☐	☐	☐	☐	
Magnetiser	☐	☐	☐	☐	☐	☐	☐	☐	☐	☐	☐	
1.3 Diabetes												
Herbal tea (to be swallowed)	☐	☐	☐	☐	☐	☐	☐	☐	☐	☐	☐	
**2. *SKIN AND EYE***												
2.1 Acne												
Aloe vera (plant to be applied)	☐	☐	☐	☐	☐	☐	☐	☐	☐	☐	☐	
Home-made mask (honey, avocado, egg, aloe vera) (to be applied)	☐	☐	☐	☐	☐	☐	☐	☐	☐	☐	☐	
2.2 Mouth ulcers / Cankers												
Honey (to be applied)	☐	☐	☐	☐	☐	☐	☐	☐	☐	☐	☐	
2.3 Spot / Pimple (diverse)												
Toothpaste (to be applied)	☐	☐	☐	☐	☐	☐	☐	☐	☐	☐	☐	
2.4 Fiver blister												
Bicarbonate (to be applied)	☐	☐	☐	☐	☐	☐	☐	☐	☐	☐	☐	
2.5 Ripening a spot (pimple)												
Cooked onion cataplasm (to be applied)	☐	☐	☐	☐	☐	☐	☐	☐	☐	☐	☐	
2.6 Burn												
Lily flower (plant to be applied)	☐	☐	☐	☐	☐	☐	☐	☐	☐	☐	☐	
Cold water rinse (to be applied)	☐	☐	☐	☐	☐	☐	☐	☐	☐	☐	☐	
Toothpaste (to be applied)	☐	☐	☐	☐	☐	☐	☐	☐	☐	☐	☐	
Potato (to be applied)	☐	☐	☐	☐	☐	☐	☐	☐	☐	☐	☐	
Butter (to be applied)	☐	☐	☐	☐	☐	☐	☐	☐	☐	☐	☐	
Vinegar (to be applied)	☐	☐	☐	☐	☐	☐	☐	☐	☐	☐	☐	
Honey (to be applied)	☐	☐	☐	☐	☐	☐	☐	☐	☐	☐	☐	
Aloe vera (to be applied)	☐	☐	☐	☐	☐	☐	☐	☐	☐	☐	☐	
Clay (mineral to be applied)	☐	☐	☐	☐	☐	☐	☐	☐	☐	☐	☐	
Fire cutter	☐	☐	☐	☐	☐	☐	☐	☐	☐	☐	☐	
Magnetiser	☐	☐	☐	☐	☐	☐	☐	☐	☐	☐	☐	
2.7 Cicatrisation												
Aloe vera (plant to be applied)	☐	☐	☐	☐	☐	☐	☐	☐	☐	☐	☐	
2.8 Contusion (bump)												
Clay (mineral to be applied)	☐	☐	☐	☐	☐	☐	☐	☐	☐	☐	☐	
Rock bitumen (mineral to be applied)	☐	☐	☐	☐	☐	☐	☐	☐	☐	☐	☐	
Ice or cold pad (to be applied)	☐	☐	☐	☐	☐	☐	☐	☐	☐	☐	☐	
2.9 Itching												
Fire cutter	☐	☐	☐	☐	☐	☐	☐	☐	☐	☐	☐	

2. SKIN AND EYE (CONTINUED)

	Frequency of use				Effectiveness							Cost
	All of the time	Often	Sometimes	Rarely	Ineffective	Somewhat	Moderately	Very	I don't know	Lemon	Honey	€
2.10 Eczema												
Nut oil (to be applied)	☐	☐	☐	☐	☐	☐	☐	☐	☐	☐	☐	
Sulfur (mineral to be applied)	☐	☐	☐	☐	☐	☐	☐	☐	☐	☐	☐	
Magnetiser	☐	☐	☐	☐	☐	☐	☐	☐	☐	☐	☐	
2.11 Psoriasis												
Magnetiser	☐	☐	☐	☐	☐	☐	☐	☐	☐	☐	☐	
2.12 Pressure ulcer												
St John's wort (to be applied)	☐	☐	☐	☐	☐	☐	☐	☐	☐	☐	☐	
2.13 Removing a splinter												
Fleur de lys (to be applied)	☐	☐	☐	☐	☐	☐	☐	☐	☐	☐	☐	
2.14 Furuncle												
Breadcrumbs + milk (to be applied)	☐	☐	☐	☐	☐	☐	☐	☐	☐	☐	☐	
2.15 Skin cracks												
Lard (to be applied)	☐	☐	☐	☐	☐	☐	☐	☐	☐	☐	☐	
Honey (to be applied)	☐	☐	☐	☐	☐	☐	☐	☐	☐	☐	☐	
2.16 Haematoma (a bruise)												
Butter + salt (to be applied)	☐	☐	☐	☐	☐	☐	☐	☐	☐	☐	☐	
Pork fat (to be applied)	☐	☐	☐	☐	☐	☐	☐	☐	☐	☐	☐	
2.17 Haemorrhage (a cut)												
Fire cutter	☐	☐	☐	☐	☐	☐	☐	☐	☐	☐	☐	
Ground pepper (to be applied)	☐	☐	☐	☐	☐	☐	☐	☐	☐	☐	☐	
Cabbage leaf (to be applied)	☐	☐	☐	☐	☐	☐	☐	☐	☐	☐	☐	
**2.18 Skin infection**												
Lily flower (plant to be applied)	☐	☐	☐	☐	☐	☐	☐	☐	☐	☐	☐	
**2.19 Mycosis**												
Garlic + coconut oil (to be swallowed)	☐	☐	☐	☐	☐	☐	☐	☐	☐	☐	☐	
**2.20 Stye**												
Black tea (to be applied)	☐	☐	☐	☐	☐	☐	☐	☐	☐	☐	☐	
Rubbing with a coin (to be applied)	☐	☐	☐	☐	☐	☐	☐	☐	☐	☐	☐	
**2.21 Panariasis**												
Cabbage leaf (to be applied)	☐	☐	☐	☐	☐	☐	☐	☐	☐	☐	☐	
**2.22 Sting**												
Vinegar (to be applied)	☐	☐	☐	☐	☐	☐	☐	☐	☐	☐	☐	
Raw onion (to be applied)	☐	☐	☐	☐	☐	☐	☐	☐	☐	☐	☐	
Garlic (to be applied)	☐	☐	☐	☐	☐	☐	☐	☐	☐	☐	☐	
Wet earth (to be applied)	☐	☐	☐	☐	☐	☐	☐	☐	☐	☐	☐	
Herbs (to be rubbed in)	☐	☐	☐	☐	☐	☐	☐	☐	☐	☐	☐	
**2.23 Mosquito bite prevention**												
Cider vinegar (to be applied)	☐	☐	☐	☐	☐	☐	☐	☐	☐	☐	☐	
Mint, verbena, thyme, lemon, lavender (to be applied)	☐	☐	☐	☐	☐	☐	☐	☐	☐	☐	☐	
**2.24 Wart**												
Wart healer	☐	☐	☐	☐	☐	☐	☐	☐	☐	☐	☐	
Celandine juice (to be applied)	☐	☐	☐	☐	☐	☐	☐	☐	☐	☐	☐	
Black adhesive tape (to be applied)	☐	☐	☐	☐	☐	☐	☐	☐	☐	☐	☐	
Onion juice (to be applied)	☐	☐	☐	☐	☐	☐	☐	☐	☐	☐	☐	
Magnetiser	☐	☐	☐	☐	☐	☐	☐	☐	☐	☐	☐	

	Frequency of use				Effectiveness							Cost
	All of the time	Often	Sometimes	Rarely	Ineffective	Somewhat	Moderately	Very	I don't know	Lemon	Honey	€
**2. *SKIN AND EYE (CONTINUED)***												
**2.25 Sweating**												
Sage infusion (to be swallowed)	☐	☐	☐	☐	☐	☐	☐	☐	☐	☐	☐	
**2.26 Zona**												
Fire cutter	☐	☐	☐	☐	☐	☐	☐	☐	☐	☐	☐	
**2.27 Conjunctivitis**												
Cerumen/earwax in the eye (to be applied)	☐	☐	☐	☐	☐	☐	☐	☐	☐	☐	☐	
Euphrasia pad (to be applied)	☐	☐	☐	☐	☐	☐	☐	☐	☐	☐	☐	
**2.28 Red / irritated eye**												
Primrose bulb pollen (plant to be applied)	☐	☐	☐	☐	☐	☐	☐	☐	☐	☐	☐	
Chamomile pad (to be applied)	☐	☐	☐	☐	☐	☐	☐	☐	☐	☐	☐	
**3. *STOMACH, INTESTINES AND DIGESTION***												
3.1 Bloating												
Cress seeds (to be swallowed)	☐	☐	☐	☐	☐	☐	☐	☐	☐	☐	☐	
Fennel infusion (to be swallowed)	☐	☐	☐	☐	☐	☐	☐	☐	☐	☐	☐	
3.2 Constipation												
Oat flakes + oat bran (to be swallowed)	☐	☐	☐	☐	☐	☐	☐	☐	☐	☐	☐	
Prunes or plums (to be swallowed)	☐	☐	☐	☐	☐	☐	☐	☐	☐	☐	☐	
Pear (to be swallowed)	☐	☐	☐	☐	☐	☐	☐	☐	☐	☐	☐	
Ginger infusion (to be swallowed)	☐	☐	☐	☐	☐	☐	☐	☐	☐	☐	☐	
Enema with olive oil product (to be applied)	☐	☐	☐	☐	☐	☐	☐	☐	☐	☐	☐	
3.3 Diarrhoea												
Banana + grated apple (to be swallowed)	☐	☐	☐	☐	☐	☐	☐	☐	☐	☐	☐	
Green walnut jam (to be swallowed)	☐	☐	☐	☐	☐	☐	☐	☐	☐	☐	☐	
Lemon juice + olive oil (to be swallowed)	☐	☐	☐	☐	☐	☐	☐	☐	☐	☐	☐	
Carrots (to be swallowed)	☐	☐	☐	☐	☐	☐	☐	☐	☐	☐	☐	
Rice / Rice cooking water (to be swallowed)	☐	☐	☐	☐	☐	☐	☐	☐	☐	☐	☐	
1 thimbleful of whisky in the morning (to be swallowed)	☐	☐	☐	☐	☐	☐	☐	☐	☐	☐	☐	
Coke (to be swallowed)	☐	☐	☐	☐	☐	☐	☐	☐	☐	☐	☐	
Ginger infusion (to be swallowed)	☐	☐	☐	☐	☐	☐	☐	☐	☐	☐	☐	
Chewing black tea	☐	☐	☐	☐	☐	☐	☐	☐	☐	☐	☐	
Small glass of pure pastis (to be swallowed)	☐	☐	☐	☐	☐	☐	☐	☐	☐	☐	☐	
3.4 Digestion												
Fennel infusion / Fennel seeds (to be swallowed)	☐	☐	☐	☐	☐	☐	☐	☐	☐	☐	☐	
Chamomile infusion (to be swallowed)	☐	☐	☐	☐	☐	☐	☐	☐	☐	☐	☐	
Mint infusion (to be swallowed)	☐	☐	☐	☐	☐	☐	☐	☐	☐	☐	☐	
Savory infusion (to be swallowed)	☐	☐	☐	☐	☐	☐	☐	☐	☐	☐	☐	
Lemon balm infusion (to be swallowed)	☐	☐	☐	☐	☐	☐	☐	☐	☐	☐	☐	
Eau de noix (homemade liqueur) / Walnut wine (to be swallowed)	☐	☐	☐	☐	☐	☐	☐	☐	☐	☐	☐	
Bottom of a glass of pastis (to be swallowed)	☐	☐	☐	☐	☐	☐	☐	☐	☐	☐	☐	
Coke (to be swallowed)	☐	☐	☐	☐	☐	☐	☐	☐	☐	☐	☐	
Aromatic herbs (to be swallowed)	☐	☐	☐	☐	☐	☐	☐	☐	☐	☐	☐	
Flaxseed (to be swallowed)	☐	☐	☐	☐	☐	☐	☐	☐	☐	☐	☐	
Honey + lemon (to be swallowed)	☐	☐	☐	☐	☐	☐	☐	☐	☐	☐	☐	
Sovereign water + bicarbonate (to be swallowed)	☐	☐	☐	☐	☐	☐	☐	☐	☐	☐	☐	

3. STOMACH, INTESTINES AND DIGESTION (CONTINU

	Frequency of use: All of the time	Often	Sometimes	Rarely	Effectiveness: Ineffective	Somewhat	Moderately	Very	I don't know	Lemon	Honey	Cost: €
**3.5 Stomach ache**												
Hot water + lemon (to be swallowed)	☐	☐	☐	☐	☐	☐	☐	☐	☐	☐	☐	
Fennel infusion (to be swallowed)	☐	☐	☐	☐	☐	☐	☐	☐	☐	☐	☐	
Mint infusion (to be swallowed)	☐	☐	☐	☐	☐	☐	☐	☐	☐	☐	☐	
Mint alcohol (to be swallowed)	☐	☐	☐	☐	☐	☐	☐	☐	☐	☐	☐	
Coke (to be swallowed)	☐	☐	☐	☐	☐	☐	☐	☐	☐	☐	☐	
Grated apple (to be swallowed)	☐	☐	☐	☐	☐	☐	☐	☐	☐	☐	☐	
Green clay (mineral to be applied)	☐	☐	☐	☐	☐	☐	☐	☐	☐	☐	☐	
Bicarbonate (to be swallowed)	☐	☐	☐	☐	☐	☐	☐	☐	☐	☐	☐	
3.6 Belly ache												
Infusion of fennel or fennel seeds (to be swallowed)	☐	☐	☐	☐	☐	☐	☐	☐	☐	☐	☐	
Chamomile infusion (to be swallowed)	☐	☐	☐	☐	☐	☐	☐	☐	☐	☐	☐	
Water + salt + lemon + garlic + cloves (to be swallowed)	☐	☐	☐	☐	☐	☐	☐	☐	☐	☐	☐	
Black basil (to be swallowed)	☐	☐	☐	☐	☐	☐	☐	☐	☐	☐	☐	
Bay leaf infusion (to be swallowed)	☐	☐	☐	☐	☐	☐	☐	☐	☐	☐	☐	
Rosemary infusion (to be swallowed)	☐	☐	☐	☐	☐	☐	☐	☐	☐	☐	☐	
Ginger infusion (to be swallowed)	☐	☐	☐	☐	☐	☐	☐	☐	☐	☐	☐	
Coke (to be swallowed)	☐	☐	☐	☐	☐	☐	☐	☐	☐	☐	☐	
Cloves (to suck)	☐	☐	☐	☐	☐	☐	☐	☐	☐	☐	☐	
Hot water bottle (to be applied)	☐	☐	☐	☐	☐	☐	☐	☐	☐	☐	☐	
Hot bath (relaxation)	☐	☐	☐	☐	☐	☐	☐	☐	☐	☐	☐	
Massage (relaxation)	☐	☐	☐	☐	☐	☐	☐	☐	☐	☐	☐	
Bread + matches + glasses stacked on stomach (to be applied)	☐	☐	☐	☐	☐	☐	☐	☐	☐	☐	☐	
Spoonful of olive oil (to be swallowed)	☐	☐	☐	☐	☐	☐	☐	☐	☐	☐	☐	
3.7 Nausea and/or vomiting												
Ginger infusion (to be swallowed)	☐	☐	☐	☐	☐	☐	☐	☐	☐	☐	☐	
Coke (to be swallowed)	☐	☐	☐	☐	☐	☐	☐	☐	☐	☐	☐	
3.8 Anthelmintic												
Garlic necklace (to wear)	☐	☐	☐	☐	☐	☐	☐	☐	☐	☐	☐	
3.9 Hemorrhoids												
Carry 3 potatoes in one's pocket	☐	☐	☐	☐	☐	☐	☐	☐	☐	☐	☐	
**4. SLEEP AND FATIGUE**												
4.1 Insomnia												
Chamomile infusion (to be swallowed)	☐	☐	☐	☐	☐	☐	☐	☐	☐	☐	☐	
Fennel infusion (to be swallowed)	☐	☐	☐	☐	☐	☐	☐	☐	☐	☐	☐	
Orange blossom (to be swallowed)	☐	☐	☐	☐	☐	☐	☐	☐	☐	☐	☐	
Verbena infusion (to be swallowed)	☐	☐	☐	☐	☐	☐	☐	☐	☐	☐	☐	
Warm milk (to be swallowed)	☐	☐	☐	☐	☐	☐	☐	☐	☐	☐	☐	
Relaxation (physical action)	☐	☐	☐	☐	☐	☐	☐	☐	☐	☐	☐	
Soft music (relaxation)	☐	☐	☐	☐	☐	☐	☐	☐	☐	☐	☐	
Reading (relaxation)	☐	☐	☐	☐	☐	☐	☐	☐	☐	☐	☐	
Yoga (relaxation)	☐	☐	☐	☐	☐	☐	☐	☐	☐	☐	☐	

	Frequency of use				Effectiveness							Cost
	All of the time	Often	Sometimes	Rarely	Ineffective	Somewhat	Moderately	Very	I don't know	Lemon	Honey	€
**4. *SLEEP AND FATIGUE (CONTINUED)***												
**4.2 Fatigue**												
Lemon juice (to be swallowed)	☐	☐	☐	☐	☐	☐	☐	☐	☐	☐	☐	
Garlic (to be swallowed)	☐	☐	☐	☐	☐	☐	☐	☐	☐	☐	☐	
Sauna (relaxation)	☐	☐	☐	☐	☐	☐	☐	☐	☐	☐	☐	
**5. *MORALE AND STRESS***												
**5.1 Low morale, depression**												
Walk (going for a stroll) (physical action)	☐	☐	☐	☐	☐	☐	☐	☐	☐	☐	☐	
Magnetiser	☐	☐	☐	☐	☐	☐	☐	☐	☐	☐	☐	
5.2 Anxiety												
Cold shower (physical action)	☐	☐	☐	☐	☐	☐	☐	☐	☐	☐	☐	
Breathing exercises (relaxation)	☐	☐	☐	☐	☐	☐	☐	☐	☐	☐	☐	
Sage (to chew)	☐	☐	☐	☐	☐	☐	☐	☐	☐	☐	☐	
5.3 Stress												
Walk (going for a stroll) (physical action)	☐	☐	☐	☐	☐	☐	☐	☐	☐	☐	☐	
Yoga (relaxation)	☐	☐	☐	☐	☐	☐	☐	☐	☐	☐	☐	
Breathing exercises (relaxation)	☐	☐	☐	☐	☐	☐	☐	☐	☐	☐	☐	
Lemon balm infusion (to be swallowed)	☐	☐	☐	☐	☐	☐	☐	☐	☐	☐	☐	
**6. *FEVER AND IMMUNE SYSTEM***												
6.1 Fever												
Vinegar pad on the limbs (to be applied)	☐	☐	☐	☐	☐	☐	☐	☐	☐	☐	☐	
Milk pad (to be applied)	☐	☐	☐	☐	☐	☐	☐	☐	☐	☐	☐	
Thyme infusion (to be swallowed)	☐	☐	☐	☐	☐	☐	☐	☐	☐	☐	☐	
Sage infusion (to be swallowed)	☐	☐	☐	☐	☐	☐	☐	☐	☐	☐	☐	
6.2 Immune system												
Infusion of thyme + rosemary (to be swallowed)	☐	☐	☐	☐	☐	☐	☐	☐	☐	☐	☐	
Ginger (to be swallowed)	☐	☐	☐	☐	☐	☐	☐	☐	☐	☐	☐	
**7. *GENITAL AND URINARY ORGANS***												
7.1 Painful menstruation												
Putting the head lower than the pelvis (physical action)	☐	☐	☐	☐	☐	☐	☐	☐	☐	☐	☐	
Oregano infusion (to be swallowed)	☐	☐	☐	☐	☐	☐	☐	☐	☐	☐	☐	
Pastis in a glass of hot water (to be swallowed)	☐	☐	☐	☐	☐	☐	☐	☐	☐	☐	☐	
Flaxseed flour (to be applied)	☐	☐	☐	☐	☐	☐	☐	☐	☐	☐	☐	
Sage infusion (to be swallowed)	☐	☐	☐	☐	☐	☐	☐	☐	☐	☐	☐	
7.2 Urinary tract infection (cystitis)												
Apple vinegar (to be swallowed)	☐	☐	☐	☐	☐	☐	☐	☐	☐	☐	☐	
Water with lemon (to be swallowed)	☐	☐	☐	☐	☐	☐	☐	☐	☐	☐	☐	
**7.3 Prostate problems**												
Pumpkin seeds (to be swallowed)	☐	☐	☐	☐	☐	☐	☐	☐	☐	☐	☐	
7.4 Libido disorders												
Savory infusion (to be swallowed)	☐	☐	☐	☐	☐	☐	☐	☐	☐	☐	☐	

8. HEAD, BACK, MUSCLES AND JOINTS

	Frequency of use				Effectiveness							Cost
	All of the time	Often	Sometimes	Rarely	Ineffective	Somewhat	Moderately	Very	I don't know	Lemon	Honey	€
8.1 Headaches / Migraine												
Coke (to be swallowed)	☐	☐	☐	☐	☐	☐	☐	☐	☐	☐	☐	
Coffee (to be swallowed)	☐	☐	☐	☐	☐	☐	☐	☐	☐	☐	☐	
Almonds (to be swallowed)	☐	☐	☐	☐	☐	☐	☐	☐	☐	☐	☐	
Weightlessness in water (relaxation)	☐	☐	☐	☐	☐	☐	☐	☐	☐	☐	☐	
Lemon juice (to be swallowed)	☐	☐	☐	☐	☐	☐	☐	☐	☐	☐	☐	
8.2 Back pain												
Heat (hot water bottle / hot shower) (relaxation)	☐	☐	☐	☐	☐	☐	☐	☐	☐	☐	☐	
Swimming on the back (physical action)	☐	☐	☐	☐	☐	☐	☐	☐	☐	☐	☐	
Lying on the ground (physical action)	☐	☐	☐	☐	☐	☐	☐	☐	☐	☐	☐	
**8.3 Cramps**												
Marseille soap in bed (to be applied)	☐	☐	☐	☐	☐	☐	☐	☐	☐	☐	☐	
Hot clay cataplasm (to be applied)	☐	☐	☐	☐	☐	☐	☐	☐	☐	☐	☐	
Water + salt + lemon juice (to be applied)	☐	☐	☐	☐	☐	☐	☐	☐	☐	☐	☐	
Vinegar (to be applied)	☐	☐	☐	☐	☐	☐	☐	☐	☐	☐	☐	
8.4 Muscle pain												
Cabbage leaves (to be applied)	☐	☐	☐	☐	☐	☐	☐	☐	☐	☐	☐	
Hot clay cataplasm (to be applied)	☐	☐	☐	☐	☐	☐	☐	☐	☐	☐	☐	
Stretching (physical action)	☐	☐	☐	☐	☐	☐	☐	☐	☐	☐	☐	
Massages (relaxation)	☐	☐	☐	☐	☐	☐	☐	☐	☐	☐	☐	
Hot water bottle (to be applied)	☐	☐	☐	☐	☐	☐	☐	☐	☐	☐	☐	
Magnetiser	☐	☐	☐	☐	☐	☐	☐	☐	☐	☐	☐	
8.5 Joint pain												
Blueberry roots (to be applied)	☐	☐	☐	☐	☐	☐	☐	☐	☐	☐	☐	
Boiled cabbage leaves (to be applied)	☐	☐	☐	☐	☐	☐	☐	☐	☐	☐	☐	
Ice (to be applied)	☐	☐	☐	☐	☐	☐	☐	☐	☐	☐	☐	
Heat (to be applied)	☐	☐	☐	☐	☐	☐	☐	☐	☐	☐	☐	
Hot bath (relaxation)	☐	☐	☐	☐	☐	☐	☐	☐	☐	☐	☐	
Stretching (physical action)	☐	☐	☐	☐	☐	☐	☐	☐	☐	☐	☐	
Clay (to be applied)	☐	☐	☐	☐	☐	☐	☐	☐	☐	☐	☐	
Glass of water with decanted clay (to be swallowed)	☐	☐	☐	☐	☐	☐	☐	☐	☐	☐	☐	
8.6 Foot pain												
Salt water footbath (relaxation)	☐	☐	☐	☐	☐	☐	☐	☐	☐	☐	☐	
Traditional healer	☐	☐	☐	☐	☐	☐	☐	☐	☐	☐	☐	
8.7 Tendonitis												
Green clay (mineral to be applied)	☐	☐	☐	☐	☐	☐	☐	☐	☐	☐	☐	
8.8 Sprain												
Clay (mineral to be applied)	☐	☐	☐	☐	☐	☐	☐	☐	☐	☐	☐	
Cold padpad (to be applied)	☐	☐	☐	☐	☐	☐	☐	☐	☐	☐	☐	
Verbena infusion (to be swallowed)	☐	☐	☐	☐	☐	☐	☐	☐	☐	☐	☐	
Onion (to be applied)	☐	☐	☐	☐	☐	☐	☐	☐	☐	☐	☐	

	Frequency of use				Effectiveness							Cost
**8. HEAD, BACK, MUSCLES AND JOINTS (CONTINUED)**	All of the time	Often	Sometimes	Rarely	Ineffective	Somewhat	Moderately	Very	I don't know	Lemon	Honey	€
***8.9* Rheumatism**												
Nettles (plant to be applied)	☐	☐	☐	☐	☐	☐	☐	☐	☐	☐	☐	
Clay (mineral to be applied)	☐	☐	☐	☐	☐	☐	☐	☐	☐	☐	☐	
Traditional healer	☐	☐	☐	☐	☐	☐	☐	☐	☐	☐	☐	
8.10 Muscle soreness / muscle ache												
Putting a chestnut in one's pocket	☐	☐	☐	☐	☐	☐	☐	☐	☐	☐	☐	
9. NOSE, THROAT, EAR AND BRONCHI												
9.1 Sinusitis												
Rosemary infusion (to inhale)	☐	☐	☐	☐	☐	☐	☐	☐	☐	☐	☐	
Saline nasal rinse (to be applied)	☐	☐	☐	☐	☐	☐	☐	☐	☐	☐	☐	
Sage (to inhale)	☐	☐	☐	☐	☐	☐	☐	☐	☐	☐	☐	
9.2 Sore throat												
Tea / tea gargle (to be swallowed / to be applied)	☐	☐	☐	☐	☐	☐	☐	☐	☐	☐	☐	
Pomegranate skin infusion (to be swallowed)	☐	☐	☐	☐	☐	☐	☐	☐	☐	☐	☐	
Thyme infusion (to be swallowed / to be applied)	☐	☐	☐	☐	☐	☐	☐	☐	☐	☐	☐	
Sage infusion / gargle (to be swallowed / to be applied)	☐	☐	☐	☐	☐	☐	☐	☐	☐	☐	☐	
Herbal infusion (to be swallowed)	☐	☐	☐	☐	☐	☐	☐	☐	☐	☐	☐	
Lemon / lemon gargle (to be swallowed / to be applied)	☐	☐	☐	☐	☐	☐	☐	☐	☐	☐	☐	
Honey / honey gargle (to be swallowed / to be applied)	☐	☐	☐	☐	☐	☐	☐	☐	☐	☐	☐	
Ginger (to be swallowed)	☐	☐	☐	☐	☐	☐	☐	☐	☐	☐	☐	
Turmeric (to be swallowed)	☐	☐	☐	☐	☐	☐	☐	☐	☐	☐	☐	
Grog (to be swallowed)	☐	☐	☐	☐	☐	☐	☐	☐	☐	☐	☐	
Milk (to be swallowed)	☐	☐	☐	☐	☐	☐	☐	☐	☐	☐	☐	
Vinegar pad on the throat (to be applied)	☐	☐	☐	☐	☐	☐	☐	☐	☐	☐	☐	
Onion cataplasm (to be applied)	☐	☐	☐	☐	☐	☐	☐	☐	☐	☐	☐	
Silk scarf around the neck (to be applied)	☐	☐	☐	☐	☐	☐	☐	☐	☐	☐	☐	
Gargle of salt water + iodine (to be applied)	☐	☐	☐	☐	☐	☐	☐	☐	☐	☐	☐	
Honey and salt (to be swallowed)	☐	☐	☐	☐	☐	☐	☐	☐	☐	☐	☐	
Gargle of lemon + alcohol (to be applied)	☐	☐	☐	☐	☐	☐	☐	☐	☐	☐	☐	
Flaxseed flour (to be applied)	☐	☐	☐	☐	☐	☐	☐	☐	☐	☐	☐	
Mustard (to be applied)	☐	☐	☐	☐	☐	☐	☐	☐	☐	☐	☐	
9.3 Toothache												
Clove (to be applied)	☐	☐	☐	☐	☐	☐	☐	☐	☐	☐	☐	
Alcohol rub (to be applied)	☐	☐	☐	☐	☐	☐	☐	☐	☐	☐	☐	
Rup plants behind the ear (to be applied)	☐	☐	☐	☐	☐	☐	☐	☐	☐	☐	☐	
9.4 Brushing teeth, Disinfecting												
Vinegar (to be applied)	☐	☐	☐	☐	☐	☐	☐	☐	☐	☐	☐	
9.5 Gum ache												
Sage gargle (to be applied)	☐	☐	☐	☐	☐	☐	☐	☐	☐	☐	☐	
Bicarbonate mouthwash (to be applied)	☐	☐	☐	☐	☐	☐	☐	☐	☐	☐	☐	
Sage infusion / gargle (to be swallowed / to be applied)	☐	☐	☐	☐	☐	☐	☐	☐	☐	☐	☐	
Infusion of mint +wild thym (to be swallowed)	☐	☐	☐	☐	☐	☐	☐	☐	☐	☐	☐	

9. NOSE, THROAT, EAR AND BRONCHI (CONTINUED)

	Frequency of use: All of the time	Often	Sometimes	Rarely	Effectiveness: Ineffective	Somewhat	Moderately	Very	I don't know	Lemon	Honey	Cost: €
9.6 Blocked ear												
Oil (to be applied)	☐	☐	☐	☐	☐	☐	☐	☐	☐	☐	☐	
9.7 Otitis												
Olive oil (to be applied)	☐	☐	☐	☐	☐	☐	☐	☐	☐	☐	☐	
Towel around the neck (to be applied)	☐	☐	☐	☐	☐	☐	☐	☐	☐	☐	☐	
9.8 Tinnitus												
Cervical exercises (physical action)	☐	☐	☐	☐	☐	☐	☐	☐	☐	☐	☐	
9.9 Bronchitis												
Mustard cataplasm (to be applied)	☐	☐	☐	☐	☐	☐	☐	☐	☐	☐	☐	
Thyme infusion (to be swallowed)	☐	☐	☐	☐	☐	☐	☐	☐	☐	☐	☐	
Succion cups (to be applied)	☐	☐	☐	☐	☐	☐	☐	☐	☐	☐	☐	
Macerated woundwort (to be swallowed / to be applied)	☐	☐	☐	☐	☐	☐	☐	☐	☐	☐	☐	
9.10 Cough												
Root vegetable syrup (turnip, black radish, carrot) (to be swallowed)	☐	☐	☐	☐	☐	☐	☐	☐	☐	☐	☐	
Chopped onion on the bedside table (to be applied)	☐	☐	☐	☐	☐	☐	☐	☐	☐	☐	☐	
Thyme infusion (to be swallowed)	☐	☐	☐	☐	☐	☐	☐	☐	☐	☐	☐	
Ginger infusion (to be swallowed)	☐	☐	☐	☐	☐	☐	☐	☐	☐	☐	☐	
Sage infusion (to be swallowed)	☐	☐	☐	☐	☐	☐	☐	☐	☐	☐	☐	
Tea (to be swallowed)	☐	☐	☐	☐	☐	☐	☐	☐	☐	☐	☐	
Onion skin infusion (to be swallowed)	☐	☐	☐	☐	☐	☐	☐	☐	☐	☐	☐	
Hot milk (to be swallowed)	☐	☐	☐	☐	☐	☐	☐	☐	☐	☐	☐	
Honey (to be swallowed)	☐	☐	☐	☐	☐	☐	☐	☐	☐	☐	☐	
Onion / onion syrup (to be swallowed)	☐	☐	☐	☐	☐	☐	☐	☐	☐	☐	☐	
Lemon (to be swallowed)	☐	☐	☐	☐	☐	☐	☐	☐	☐	☐	☐	
Garlic (to be swallowed)	☐	☐	☐	☐	☐	☐	☐	☐	☐	☐	☐	
Elderflower syrup (to be swallowed)	☐	☐	☐	☐	☐	☐	☐	☐	☐	☐	☐	
Clay cataplasm (mineral to be applied)	☐	☐	☐	☐	☐	☐	☐	☐	☐	☐	☐	
Cooked onion cataplasm (to be applied)	☐	☐	☐	☐	☐	☐	☐	☐	☐	☐	☐	
Potato on chest (to be applied)	☐	☐	☐	☐	☐	☐	☐	☐	☐	☐	☐	
9.11 Clear the airways												
Wet cloth on the radiator (to be applied)	☐	☐	☐	☐	☐	☐	☐	☐	☐	☐	☐	
Inhalation of potato peelings (to inhale)	☐	☐	☐	☐	☐	☐	☐	☐	☐	☐	☐	
9.12 Common cold												
Honey (to be swallowed)	☐	☐	☐	☐	☐	☐	☐	☐	☐	☐	☐	
Lemon (to be swallowed)	☐	☐	☐	☐	☐	☐	☐	☐	☐	☐	☐	
Ginger (to be swallowed)	☐	☐	☐	☐	☐	☐	☐	☐	☐	☐	☐	
Turmeric pepper (to be swallowed)	☐	☐	☐	☐	☐	☐	☐	☐	☐	☐	☐	
Root syrup (to be swallowed)	☐	☐	☐	☐	☐	☐	☐	☐	☐	☐	☐	
Royal jelly (to be swallowed)	☐	☐	☐	☐	☐	☐	☐	☐	☐	☐	☐	

Garlic (to be swallowed)	☐	☐	☐	☐	☐	☐	☐	☐	☐	☐	☐	
Onion / onion juice (to be swallowed)	☐	☐	☐	☐	☐	☐	☐	☐	☐	☐	☐	

9. NOSE, THROAT, EAR AND BRONCHI (CONTINUED)

	Frequency of use				Effectiveness							Cost
	All the time	Often	Sometimes	Rarely	Ineffective	Litle	Moderately	Very	I don’t knowl	Lemon	Honey	€
9.12 Common cold (CONTINUED)												
Black berry leaves (to be swallowed / infusion)	☐	☐	☐	☐	☐	☐	☐	☐	☐	☐	☐	
Thyme infusion (to be swallowed)	☐	☐	☐	☐	☐	☐	☐	☐	☐	☐	☐	
Rosemary infusion (to be swallowed)	☐	☐	☐	☐	☐	☐	☐	☐	☐	☐	☐	
Sage infusion (to be swallowed)	☐	☐	☐	☐	☐	☐	☐	☐	☐	☐	☐	
Ginger infusion (to be swallowed)	☐	☐	☐	☐	☐	☐	☐	☐	☐	☐	☐	
Chamomile infusion (to be swallowed)	☐	☐	☐	☐	☐	☐	☐	☐	☐	☐	☐	
Infusion of cinnamon + clove (to be swallowed)	☐	☐	☐	☐	☐	☐	☐	☐	☐	☐	☐	
Mountain herb infusion (to be swallowed)	☐	☐	☐	☐	☐	☐	☐	☐	☐	☐	☐	
Onion skin infusion (to be swallowed)	☐	☐	☐	☐	☐	☐	☐	☐	☐	☐	☐	
Herbal infusion (to be swallowed)	☐	☐	☐	☐	☐	☐	☐	☐	☐	☐	☐	
Tea (to be swallowed)	☐	☐	☐	☐	☐	☐	☐	☐	☐	☐	☐	
Onion cut near the bed (to be applied)	☐	☐	☐	☐	☐	☐	☐	☐	☐	☐	☐	
Grog (to be swallowed)	☐	☐	☐	☐	☐	☐	☐	☐	☐	☐	☐	
Hot milk (to be swallowed)	☐	☐	☐	☐	☐	☐	☐	☐	☐	☐	☐	
Fresh orange juice (to be swallowed)	☐	☐	☐	☐	☐	☐	☐	☐	☐	☐	☐	
Orange juice with a few drops of eau de vie (to be swallowed)	☐	☐	☐	☐	☐	☐	☐	☐	☐	☐	☐	
Sugar + eau de vie (to be swallowed)	☐	☐	☐	☐	☐	☐	☐	☐	☐	☐	☐	
Thyme inhalation (to inhale)	☐	☐	☐	☐	☐	☐	☐	☐	☐	☐	☐	
Bay leaf inhalation (to inhale)	☐	☐	☐	☐	☐	☐	☐	☐	☐	☐	☐	
Hot water inhalation (to inhale)	☐	☐	☐	☐	☐	☐	☐	☐	☐	☐	☐	
Eucalyptus leaf inhalation (to inhale)	☐	☐	☐	☐	☐	☐	☐	☐	☐	☐	☐	
Ignite eucalyptus leaves	☐	☐	☐	☐	☐	☐	☐	☐	☐	☐	☐	
Sauna (relaxation)	☐	☐	☐	☐	☐	☐	☐	☐	☐	☐	☐	
Garlic in one nostril (to be applied)	☐	☐	☐	☐	☐	☐	☐	☐	☐	☐	☐	
Salt in the nose (to be applied)	☐	☐	☐	☐	☐	☐	☐	☐	☐	☐	☐	
Lemon in the nose (to be applied)	☐	☐	☐	☐	☐	☐	☐	☐	☐	☐	☐	
Potato on the chest (to be applied)	☐	☐	☐	☐	☐	☐	☐	☐	☐	☐	☐	
Pectoral plants (violet, cuckoo)	☐	☐	☐	☐	☐	☐	☐	☐	☐	☐	☐	
9.13 Pollen allergy												
Thyme infusion (to be swallowed)	☐	☐	☐	☐	☐	☐	☐	☐	☐	☐	☐	
Fresh ginger (to be swallowed)	☐	☐	☐	☐	☐	☐	☐	☐	☐	☐	☐	

10. OTHER

10.1 Cancer-related pain												
Fire cutter	☐	☐	☐	☐	☐	☐	☐	☐	☐	☐	☐	
Magnetiser	☐	☐	☐	☐	☐	☐	☐	☐	☐	☐	☐	
10.2 Elimination of toxins / Cleansing the liver												
Hot water + lemon (to be swallowed)	☐	☐	☐	☐	☐	☐	☐	☐	☐	☐	☐	
Vinegar (to be swallowed)	☐	☐	☐	☐	☐	☐	☐	☐	☐	☐	☐	

	Frequency of use				Effectiveness							Cost
	All the time	Often	Sometimes	Rarely	Ineffective	Litle	Moderately	Very	I don’t knowl	Lemon	Honey	
Honey (to be swallowed)		☐		☐		☐		☐		☐		
Artichoke infusion (to be swallowed)		☐		☐		☐		☐		☐		
Herbal tea (to be swallowed)		☐		☐		☐		☐		☐		€
10. OTHER (CONTINUED)												
10.3 In "general" prevention												
Lemon juice in the morning (to be swallowed)	☐	☐	☐	☐	☐	☐	☐	☐	☐	☐	☐	
A glass of eau de vie every morning (to be swallowed)	☐	☐	☐	☐	☐	☐	☐	☐	☐	☐	☐	
Milk with honey in the morning (to be swallowed)	☐	☐	☐	☐	☐	☐	☐	☐	☐	☐	☐	
10.4 Antibacterial												
Honey (to be swallowed)	☐	☐	☐	☐	☐	☐	☐	☐	☐	☐	☐	
Thyme (to be swallowed)	☐	☐	☐	☐	☐	☐	☐	☐	☐	☐	☐	
11. HAVE YOU USED ANY OTHER HOME REMEDIES? IF SO, WHICH ONES?	☐	☐	☐	☐	☐	☐	☐	☐	☐	☐	☐	
	☐	☐	☐	☐	☐	☐	☐	☐	☐	☐	☐	
	☐	☐	☐	☐	☐	☐	☐	☐	☐	☐	☐	
	☐	☐	☐	☐	☐	☐	☐	☐	☐	☐	☐	
	☐	☐	☐	☐	☐	☐	☐	☐	☐	☐	☐	
	☐	☐	☐	☐	☐	☐	☐	☐	☐	☐	☐	
